# Prospective Study of Sex-Specific Adiponectin Changes and Incident Metabolic Syndrome: The ARIRANG Study

**DOI:** 10.3390/jcm8050599

**Published:** 2019-05-01

**Authors:** Ji Hye Huh, Tae Woong Yoon, Dae Ryong Kang, Jang Young Kim

**Affiliations:** 1 Department of Internal Medicine, Wonju College of Medicine, Yonsei University, Wonju 26426, Korea; png1212@yonsei.ac.kr; 2 Department of Biostatistics, Wonju College of Medicine, Yonsei University, Wonju 26426, Korea; ytw31246@gmail.com (T.W.Y.); dr.kang@yonsei.ac.kr (D.R.K.); 3 Center of Biomedical Data Science/Institute of Genomic Cohort, Wonju College of Medicine, Yonsei University, Wonju 26426, Korea

**Keywords:** adiponectin change, metabolic syndrome, sex difference

## Abstract

We investigated whether changes in adiponectin levels over time predict incident metabolic syndrome (MetS) in a population-based prospective study. In total, 1110 subjects were categorized into four groups according to their sex-specific median baseline adiponectin levels and the change in adiponectin levels at follow-up: low baseline adiponectin and decreased adiponectin during follow-up (LB&DF), low baseline adiponectin and increased adiponectin during follow-up (LB&IF), high baseline adiponectin and decreased adiponectin during follow-up (HB&DF), and high baseline adiponectin and increased adiponectin during follow-up (HB&IF). During the median 2.4-year follow-up period, 180 (16.2%) subjects developed MetS. Compared to the LB&DF group, the fully adjusted hazard ratio (95% confidence interval) for incident MS was the lowest in the HB&IF group (0.33, (0.17–0.63)), followed by the HB&DF group (0.58, (0.40–0.84)) and LB&IF group (0.63, (0.41–0.93)). This phenomenon was more prominent in men than in women. Among the individual MetS components, increased adiponectin levels during follow-up were significantly associated with lower risks of incident low high density lipoprotein (HDL) cholesterol and incident high blood pressure. This finding suggests that a change in adiponectin level, as well as the baseline adiponectin level, might have a clinical role in the development of MetS among men.

## 1. Introduction

Metabolic syndrome (MetS) refers to a cluster of cardiovascular disease risk factors whose underlying pathophysiology is related to insulin resistance; these factors include central obesity, dyslipidemia, impaired glucose tolerance, and hypertension [1]. MetS is a disorder with growing prevalence [2], and it is a well-recognized risk factor for cardiovascular disease [3], diabetes, and chronic kidney disease [4]. Given the high prevalence of metabolic syndrome and its potential consequences, there is substantial interest in understanding its causes and mechanisms using population-based longitudinal studies. 

MetS is closely linked to obesity and adipose tissue dysfunction. Dysfunctional adipose tissue secretes signaling molecules and hormones, which are collectively termed adipokines. Adiponectin, one of the adipokines exclusively secreted from adipose tissue, has protective properties against obesity-related diseases [5]. Although adiponectin is secreted from adipose tissue, expression of adiponectin and its receptor are decreased in subjects with obesity or metabolic abnormalities [6]. A recent study found that in obese subjects, weight loss is correlated with a rise in adiponectin levels, with a specific increase of the most biologically active form [7]. This suggests a functional recovery in adipose tissue after lifestyle modifications that consequently induces an increase in adiponectin secretion. From these results, we can hypothesize that increasing adiponectin levels can reduce the risk of MetS. 

Although previous studies have reported that higher baseline adiponectin levels are protective against incident MetS [8], little is known about the interrelationship between baseline adiponectin levels, changes in adiponectin levels, and the development of MetS. Moreover, while there are considerable differences in circulating adiponectin levels among men and women, few studies have investigated the sex-specific effects of circulating adiponectin levels on health outcomes. To address these issues, we investigated the impact of changes in serum adiponectin levels on the risk of metabolic syndrome in relatively healthy Korean men and women. Additionally, we assessed the relative contributions of baseline adiponectin levels and changes in adiponectin levels during follow-up to the development of MetS considering sex-specific differences using a large cohort study.

## 2. Methods

### 2.1. Subjects

The present study used data from the Korean Genome and Epidemiology Study on Atherosclerosis Risk of Rural Areas in the Korean General Population (KoGES-ARIRANG), a population-based prospective cohort study to assess the prevalence, incidence, and risk factors for chronic degenerative disorders such as hypertension, diabetes, and cardiovascular disease [9]. KoGES-ARIRANG invited all adults aged 40–70 years who resided in rural areas of Wonju and Pyeongchang in South Korea to participate in the study. Demographic shifts are infrequent in this area, and the population can be followed long term [10]. The baseline survey was carried out from November 2005 to January 2008 in adults aged 40–70 years. All study participants were invited to the first follow-up survey from April 2008 to January 2011. Among these participants, we included 1110 subjects (458 men and 652 women) for whom data on baseline adiponectin levels, follow-up, and MetS were available. Median follow-up period was 2.4 years (interquartile range 2.2–2.8). The subjects were categorized into four groups according to their sex-specific median adiponectin levels at the baseline and their change in adiponectin level at follow-up: (1) low adiponectin at baseline and decreased adiponectin during follow-up (*n* = 334); (2) low adiponectin at baseline and increased adiponectin during follow-up (*n* = 221); (3) high adiponectin at baseline and decreased adiponectin during follow-up (*n* = 401); and (4) high adiponectin at baseline and increased adiponectin during follow-up (*n* = 154). At each visit, informed written consent was obtained from all participants. The study protocol was approved by the Ethics Committee of the Korean Center for Disease Control and the Institutional Review Boards of the Yonsei University Wonju College of Medicine (YWMR-15-9-075).

### 2.2. Data Collection

At the baseline and follow-up examinations, study participants completed a standardized medical history and lifestyle questionnaire and underwent a comprehensive health examination according to standard procedures. Body weight and height were measured while participants were wearing light indoor clothing without shoes. Blood pressure was measured from the right arm after the participant had rested for at least 5 min in a quiet room using a standard mercury sphygmomanometer (Baumanometer, Copiague, NY, USA). Two measurements were made with at least 5 min intervals in between, and the mean of the two measurements was used in the analyses. Smoking status and exercise level were determined based on self-reports. Current smokers were defined as participants who had smoked ≥100 cigarettes in their lifetime and who reported “currently smoking”’ in the questionnaire. Subjects who answered “yes” to the question “Do you perform physical exercise regularly enough to make you sweat?” were assigned to the regular exercise group. A venous blood sample was drawn from study participants after fasting for ≥12 h or overnight. Serum aliquots were centrifuged then stored at −80℃ until thawed for adiponectin analysis within one week after centrifugation. The serum concentrations of adiponectin were measured using radioimmunoassays (RIAs) (LINCO Research, Inc., St.Charles, MO, SA) with intra-assay and inter-assay coefficients of variation ranging between 2.9% and 6.6%. Fasting glucose was determined using a glucose oxidase-based assay. The serum concentrations of total cholesterol, high density lipoprotein (HDL) cholesterol, and triglycerides (TG) were measured using enzymatic methods (Advia 1650, Siemens, Tarrytown, NY, USA). High sensitivity C-reactive protein (hs-CRP) was measured using the Denka Seiken (Tokyo, Japan) assay, which has been validated against the Dade Behring method. The homeostasis model assessment for insulin resistance (HOMA-IR) levels were calculated using the following formula: fasting plasma insulin (μ IU/mL) × fasting plasma glucose (mg/dL)/405.

### 2.3. Definition of Incident Metabolic Syndrome

The study endpoint was incident MetS at the follow-up visit. The definition of MetS in this study was based on a modification of the National Cholesterol Education Program Adult Treatment Panel III (NCEP-ATP III) criteria [11]. MetS was defined as the presence of three or more of the following: (1) abdominal obesity, defined as a waist circumference ≥90 cm for males or ≥85 cm for females according to the cutoffs for abdominal obesity defined by the Korean Society of Obesity [12]; (2) hypertriglyceridemia, defined as a serum TG concentration ≥150 mg/dL; (3) low HDL cholesterol, defined as a serum HDL cholesterol concentration <40 mg/dL for males or <50 mg/dL for females; (4) high blood pressure, defined as systolic blood pressure (SBP) ≥130 mmHg, diastolic blood pressure (DBP) ≥85 mmHg, or treatment with antihypertensive agents; and (5) high fasting glucose, defined as a fasting serum glucose ≥100 mg/dL. 

### 2.4. Statistical Analysis

Since the women had higher levels of adiponectin compared to the men, we performed all analyses separately for men and women. The distributions of serum TG, adiponectin, hs-CRP, and HOMA-IR scores were markedly skewed; therefore, they were log-transformed for the analyses where normality was required. We divided the study population according to the median baseline serum adiponectin level with sex-specific cut-offs of 9.31 mg/mL for men and 13.62 mg/mL for women. Additionally, we divided participants into two categories according to the change in adiponectin level at the follow-up. Differences among the groups were analyzed using analysis of variance (ANOVA) with the Bonferroni post hoc analysis method for continuous variables and the chi-squared test for categorical variables. Cox proportional hazards analyses were conducted to estimate the hazard ratio (HR) and 95% confidence interval (CI) for the association between adiponectin levels and the development of MetS. A *p*-value of <0.05 was considered statistically significant, and SAS software, version 9.2 (SAS Inc., Cary, NC, USA) was used to conduct all analyses. 

## 3. Results

### 3.1. Baseline Characteristics 

The baseline characteristics of the study participants according to the baseline and change in adiponectin levels are presented in Table 1 and Table S1. There were 334 (30%) subjects with low baseline adiponectin and decreased adiponectin during follow-up (LB&DF), 221 (19.9%) with low baseline adiponectin and increased adiponectin during follow-up (LB&IF), 401 (36.1%) with high baseline adiponectin and decreased adiponectin during follow-up (HB&DF), and 154 (13.9%) with high baseline adiponectin and increased adiponectin during follow-up (HB&IF). Compared to subjects with low adiponectin levels at the baseline, subjects with higher baseline adiponectin levels were older and had lower body mass index (BMI), lower waist circumference, lower HOMA-IR levels, and higher HDL cholesterol. Moreover, the proportion of current smokers was lower in subjects with higher adiponectin at the baseline. Compared to subjects with a decreased level of adiponectin at the follow up, subjects with an increased level of adiponectin during the same period had lower waist circumferences, lower TG levels, lower albumin levels, and lower HOMA-IR scores. Serum adiponectin level at the follow-up was highest in the HB&IF group, followed by the HB&DF group, the LB&IF group, and the LB&DF group, in that order (*p* < 0.001). However, there were no significant differences in follow-up adiponectin levels between the LB&IF group and HB&DF group. The proportions of regular exercise were not different among four groups.

#### 3.1.1. The Risk of Incident MetS According to the Baseline Adiponectin Level and the Change in Adiponectin Level

Table 2 shows the results of the Cox proportional-hazards analyses for incident MetS categorized according to the sex-specific median adiponectin levels at the baseline and the change in adiponectin level at the follow-up. During a median 2.4 years (interquartile range (IQR), 2.2–2.8) of follow-up, new onset MetS developed in 71 men (15.5%) and 109 women (16.7%). Using the LB&DF group as a reference, the risk of incident MetS gradually decreased in the HB&IF group followed by the HB&DF group and the LB&IF group, in that order. After adjusting for age, sex, smoking status, regular exercise, alcohol intake, baseline BMI, follow-up BMI, and baseline hs-CRP, LDL cholesterol, and HOMA-IR, this trend remained significant with a fully adjusted HR (95% CI) for new-onset MetS of 0.33 (0.17–0.63) in the HB&IF group, 0.58 (0.40–0.84) in the HB&DF group, and 0.62 (0.41–0.93) in the LB&IF group (Table 2, Total Model 2). When the analysis was stratified by sex, this trend was consistently seen in men even after adjusting for confounding factors (Men Model 2). However, the independent association between the risk of incident MetS and adiponectin levels was not significant in women (*p* = 0.10).

#### 3.1.2. The Incident Risk of Individual MetS Components According to the Baseline Adiponectin Level and the Change in Adiponectin Level

**Figure 1:**
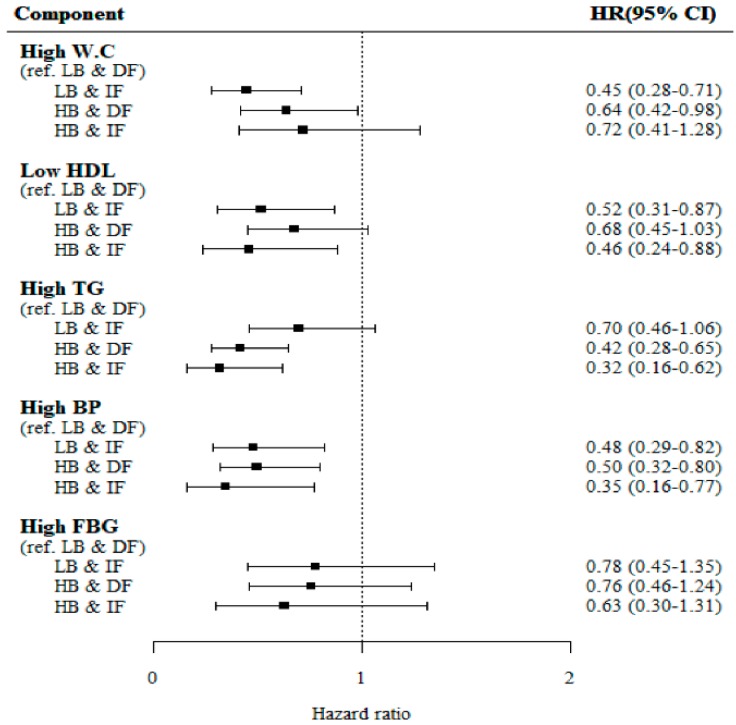
Association between adiponectin concentrations and incidence for each component of metabolic syndrome. Adjustment for age, sex, current smoking, regular exercise, alcohol intake, baseline body mass index, high sensitive C-reactive protein, low density protein lipoprotein cholesterol, HOMA-IR (log transformed), and follow-up body mass index. HR, hazard ratio; W.C, waist circumference; HDL, high density lipoprotein cholesterol; TG, triglyceride; BP, blood pressure; FBG, fasting blood glucose; LB&DF, low adiponectin at baseline and decreased adiponectin during follow-up; LB&IF, low adiponectin at baseline and increased adiponectin during follow-up; HB&DF, high adiponectin at baseline and decreased adiponectin during follow-up; HB&IF, high adiponectin at baseline and increased adiponectin during follow-up.

**Figure 2:**
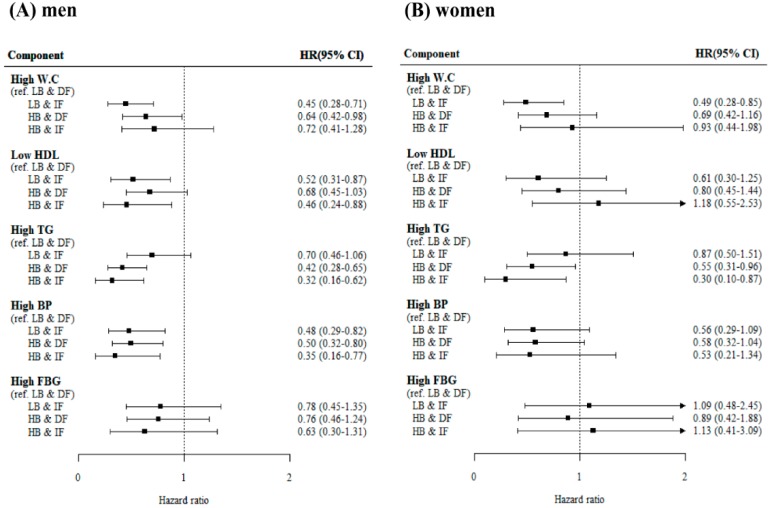
Association between adiponectin concentrations and incidence of each component of metabolic syndrome stratified by sex (men (**A**) and women (**B**)). Adjustment for age, current smoking, regular exercise, alcohol intake, baseline body mass index, high sensitive C-reactive protein, low density protein lipoprotein cholesterol, HOMA-IR (log transformed), and follow-up body mass index. W.C, waist circumference; HDL, high density lipoprotein cholesterol; TG, triglyceride; BP, blood pressure; FBG, fasting blood glucose; LB&DF, low adiponectin at baseline and decreased adiponectin during follow-up; LB&IF, low adiponectin at baseline and increased adiponectin during follow-up; HB&DF, high adiponectin at baseline and decreased adiponectin during follow-up; HB&IF, high adiponectin at baseline and increased adiponectin during follow-up.

Figure 1 shows the incident risk for each MetS component among the four groups. After adjusting for age, sex, smoking status, regular exercise, alcohol intake, baseline BMI, follow-up BMI, and baseline hs-CRP, low density lipoprotein cholesterol, and HOMA-IR, the adjusted HRs for incident high waist circumference and incident high glucose were not significantly different among the four groups. However, compared with the reference group (LB&DF), the risk of incident low HDL cholesterol was the lowest in the HB&IF group followed by the LB&IF group and the HB&DF group, in that order (*p* for trend <0.001). The risk of incident high TG was the lowest in the HB&IF group followed by the HB&DF group and the LB&DF group, in that order (*p* for trend = 0.002). The risk of incident high blood pressure was the lowest in the HB&IF group followed by the LB&IF group and the HB&DF group, in that order (*p* for trend = 0.002). When the analysis was stratified by sex (Figure 2), the results and patterns observed in men were similar to those for the total subject population. However, no significant differences were found in the risk of metabolic abnormalities for women except incident high TG. The risk of incident high TG was the lowest in the in the HB&IF group followed by the HB&DF group and the LB&DF group, in that order (*p* for trend = 0.002).

## 4. Discussion

In this longitudinal cohort study in a healthy Korean population, we demonstrated that an increase in adiponectin level over the 2.4-year follow-up period was significantly associated with a lower risk of developing MetS. We also found that the lower risk of incident MetS in subjects with increased adiponectin levels during follow-up was significantly independent of baseline adiponectin levels. Moreover, we observed that the association between changes in adiponectin levels and the risk of developing MS was more prominent in men than in women. To the best of our knowledge, this is the first population-based study of the relationship between changes in adiponectin level and MetS that considers the sex-specific effect of circulating adiponectin. 

Adiponectin has beneficial effects on glucose homeostasis, chronic low-grade inflammation, oxidative stress, and atherosclerotic processes, so this molecule has usually been considered a salutary adipokine [13]. Paradoxically, recent epidemiological studies have reported that higher adiponectin concentration at the baseline was associated with high mortality, particularly cardiovascular mortality in frail individuals at a high mortality risk [14,15]. Due to the debate regarding the effect of circulating adiponectin levels on cardio-metabolic health in humans, more confirmative studies have been needed to determine the direct role of circulating adiponectin in health outcomes. Recent studies have concluded that the observed deleterious effects of adiponectin on the risk of mortality are due to natriuretic peptides, an established risk factor of mortality as well as increased factor in critically ill patients [16]. Natriuretic peptides stimulate the secretion of adiponectin; therefore, natriuretic peptides are the underlying risk factor of mortality with adiponectin being only a marker of increased natriuretic peptide levels. However, our study was performed in relatively healthy subjects to minimize the effects of possible confounders or modulators. Furthermore, to elucidate the interrelationship between baseline circulating adiponectin levels, changes in adiponectin levels, and the development of MetS, we categorized the subjects into four groups according to the baseline adiponectin levels and the adiponectin level change during the follow-up period. Our findings further support the conclusion that circulating adiponectin has protective effects against metabolic disorders in the healthy population.

The other principal finding of our study is the sex-specific association between circulating adiponectin levels and incident MetS with the salutary effect of increased adiponectin levels during follow-up being observable only among men. Contradictory findings have previously been reported regarding a sexually dimorphic association of adiponectin with metabolic disease [17,18]. To investigate whether circulating adiponectin plays a role in the development of MetS in a sex-specific manner, the analysis of serum adiponectin levels was stratified by sex in our study. We found that high circulating adiponectin levels at the baseline and increased adiponectin levels during the follow-up predicted a lower risk of developing MetS in men but not in women. While the previous studies reporting a sexually dimorphic association of adiponectin with metabolic disease only investigated the effects of circulating adiponectin measured at baseline, our finding clearly demonstrated that adiponectin changes also have a different relationship to incident MetS especially in men. However, this observational study could not elucidate the mechanism of the sex-specific association between adiponectin level and incident MetS. Although some researchers have suggested that different adiponectin bioactivity could be an explanation for the sex-specific finding [19], further studies regarding the interaction between circulating adiponectin and either sex-linked genes and/or sexual hormone effects are needed in the future. 

Our study has several limitations. First, we measured only the total adiponectin level. Adiponectin is secreted from adipocytes into the bloodstream in three oligomeric complexes: trimers, hexamers, and high molecular weight (HMW) multimers comprising at least 18 monomers. Generally, HMW adiponectin is responsible for the majority of the insulin sensitizing effects [20]. However, several previous studies have demonstrated that the associations of both total adiponectin and HMW adiponectin with metabolic disease are similar [21,22]. Second, our analyses were based on a single determination of serum adiponectin, which is subject to random measurement error and may have underestimated the strength of the associations. In addition, the effects of potential biases affecting adiponectin levels (including polypharmacy) and other unmeasured confounding factors not taken into account in our study cannot be excluded. Third, due to the short follow-up period of our cohort (median 2.4 years), we could not evaluate the association between adiponectin level changes and other metabolic disease such as diabetes or cardiovascular disease. Fourth, although we observed that leptin to adiponectin ratio and leptin level at baseline was higher in incident MetS group (Table S2), we could not analyze the association between change of leptin to adiponectin ratio (which is well known marker of insulin resistance) and incident MetS due to missing data on leptin levels at follow-up. Finally, because this study was performed in a rural Korean population and sample size is too small, our findings may not be generalizable to other populations.

## 5. Conclusions 

In summary, this longitudinal study performed in a healthy, rural, Korean population revealed that changes in circulating adiponectin level over 2.4 years of follow-up, as well as adiponectin levels at the baseline, were independently associated with a lower risk of MetS. When the analysis was stratified by sex, these associations were only observed in men, whereas no associations regarding developing MetS or majority of its components were detected in women. These results suggest that high or increased serum adiponectin levels might play a protective role against metabolic disease especially in men. Additional long-term prospective studies in other populations, including subjects with various other health conditions, are needed to support our conclusion that increased adiponectin levels, as well as high baseline adiponectin levels, independently lower the risk of incident MetS.

## Figures and Tables

**Table 1:** Baseline characteristics of participants according to median of plasma adiponectin at baseline and change of plasma adiponectin during follow up period.

	Low Adiponectin at Baseline		High Adiponectin at Baseline		
Variable	Decreased Adiponectin during Follow-Up (*n* = 334)	Increased Adiponectin during Follow-Up (*n* = 221)	Decreased Adiponectin during Follow-Up (*n* = 401)	Increased Adiponectin during Follow-Up (*n* = 154)	*p*-Value
**Age (years)**	53.01 ± 7.86 ^‡§^	54.70 ± 7.95	55.71 ± 8.47 *	56.71 ± 8.13 *	<0.0001
**Sex (male, %)**	136 (40.72)	93 (42.08)	169 (42.14)	60 (38.96)	0.904
**BMI (kg/m^2^)**	24.00 ± 2.74 ^§^	23.80 ± 2.65	23.63 ± 2.81	23.09 ± 3.04 *	0.009
**BMI change**	−0.32 ± 1.19 ^†^	0.00 ± 1.18 *^‡^	−0.29 ± 1.12 ^†^	−0.25 ± 1.22	0.008
**Waist circumference**	81.38 ± 7.46 ^§^	80.11 ± 7.53	80.96 ± 7.89	79.28 ± 8.36 *	0.026
**Systolic BP (mmHg)**	121.28 ± 16.26	123.48 ± 16.26	123.74 ± 16.35	124.96 ± 16.29	0.075
**Diastolic BP (mmHg)**	75.56 ± 10.64	80.45 ± 11.19	80.67 ± 11.06	81.97 ± 11.08	0.151
**hsCRP (mg/L)**	1.91 ± 4.69	2.01 ± 6.63	1.96 ± 4.34	2.50 ± 10.85	0.788
**BUN (mg/dL)**	15.32 ± 4.05	14.77 ± 4.00 ^‡§^	15.74 ± 4.15 ^†^	15.97 ± 4.73 ^†^	0.016
**Cr (mg/dL)**	0.95 ± 0.15	0.94 ± 0.14	0.94 ± 0.16	0.96 ± 0.20	0.623
**Fasting glucose (mg/dL)**	91.78 ± 18.31	90.47 ± 10.83	90.12 ± 12.44	89.66 ± 15.57	0.356
**Total cholesterol (mg/dL)**	200.51 ± 35.55	196.06 ± 35.66	202.75 ± 36.23	198.43 ± 37.18	0.151
**HDL-C (mg/dL)**	47.89 ± 9.92 ^‡^	48.52 ± 10.58 ^‡^	51.90 ± 12.49 *^†^	50.55 ± 10.36	<0.0001
**LDL-C (mg/dL)**	117.77 ± 30.12	117.35 ± 29.91	117.89 ± 30.77	117.73 ± 32.33	0.997
**Triglyceride (mg/dL)**	107.5 (81.0–143.0) ^§^	99.0 (75.0–129.0)	102.0 (77.0–133.0)	94.0 (71.0–122.0) *	0.001
**Albumin (g/dL)**	4.66 ± 0.26 ^†§^	4.58 ± 0.25 *	4.62 ± 0.27 ^§^	4.54 ± 0.25 *^‡^	<0.0001
**Adiponectin (baseline)**	7.20 (5.45–8.66) ^‡§^	6.25 (4.87–7.90) ^‡§^	13.65 (11.68–16.61) *^†^	13.01 (11.35–15.31) *^†^	<0.0001
**Adiponectin (follow-up)**	5.23 (3.60–6.94) ^†‡§^	8.89 (6.66–11.89) *^§^	9.75 (7.30–12.20) *^§^	15.54 (13.43–18.76) *^†‡^	<0.0001
**Leptin**	4.61 (2.30–8.54)	4.87 (1.81–8.18)	4.00 (1.92–7.68)	3.96 (1.94–6.89)	0.106
**HOMA-IR**	1.54 (1.24–1.98) ^§^	1.48 (1.21–1.89)	1.49 (1.20–1.89)	1.34 (1.08–1.75) *	0.006
**Alcohol intake (%)**	155 (46.41)	85 (38.46)	185 (46.13)	62 (40.26)	0.161
**Regular exercise (%)**	90 (27.27)	60 (27.15)	103 (25.88)	38 (24.68)	0.922
**Current smoker (%)**	72 (21.56)	37 (16.74)	65 (16.21)	17 (11.04)	0.031

BMI, body mass index; BP, blood pressure; hsCRP, high sensitive C-reactive protein; BUN, blood urea nitrogen; Cr, creatinine; HDL-C, high density lipoprotein cholesterol; LDL-C, low density lipoprotein cholesterol; HOMA-IR, homeostasis model assessment for insulin resistance. * *p* < 0.05 vs. low adiponectin at baseline and decreased adiponectin during follow-up; ^†^ *p* < 0.05 vs. low adiponectin at baseline and increased adiponectin during follow-up; ^‡^ *p* < 0.05 vs. high adiponectin at baseline and decreased adiponectin during follow-up; ^§^ *p* < 0.05 vs. high adiponectin at baseline and increased adiponectin during follow-up.

**Table 2:** Association between adiponectin concentrations and incidence of metabolic syndrome.

	Low Baseline Decreased FU	Low Baseline Increased FU	High Baseline Decreased FU	High Baseline Increased FU	*p* for Trend
Total (*n* = 1110)	334	221	401	154	
Number of incident case (%)	61 (18.26)	51 (23.08)	56 (13.97)	12 (7.79)	0.001
Person-years	880.83	759.08	1188.83	422.25	
Incidence rate (1000 person-year)	69.25	67.19	47.11	28.42	
Crude HR	reference	0.79 (0.54–1.15)	0.59 (0.41–0.85)	0.41 (0.22–0.77)	<0.0001
Model 1	reference	0.76 (0.52–1.11)	0.55 (0.38–0.80)	0.38 (0.20–0.71)	<0.0001
Model 2	reference	0.62 (0.41–0.93)	0.58 (0.40–0.84)	0.33 (0.17–0.63)	<0.0001
Men (*n* = 458)	136	93	169	60	
Number of incident cases (%)	25 (18.38)	22 (23.66)	20 (11.83)	4 (6.67)	0.010
Person-years	357.08	318.17	475.67	183.50	
Incidence rate (1000 person-year)	70.01	69.15	42.05	21.80	
Crude HR	reference	0.70 (0.39–1.27)	0.52 (0.29–0.93)	0.25 (0.09–0.73)	0.002
Model 1	reference	0.65 (0.35–1.18)	0.46 (0.25–0.86)	0.21 (0.07–0.64)	0.001
Model 2	reference	0.39 (0.19–0.78)	0.36 (0.18–0.69)	0.08 (0.02–0.27)	<0.0001
Women (*n* = 652)	198	128	232	94	
Number of incident case (%)	36 (18.18)	29 (22.66)	36 (15.52)	8 (8.51)	0.036
Person-years	523.75	440.92	713.17	238.75	
Incident rate (1000 person-year)	68.74	65.77	50.48	33.51	
Crude HR	reference	0.84 (0.51–1.39)	0.65 (0.41–1.03)	0.55 (0.26–1.19)	0.033
Model 1	reference	0.81 (0.49–1.34)	0.59 (0.37–0.94)	0.51 (0.23–1.09)	0.012
Model 2	reference	0.73 (0.43–1.23)	0.65 (0.40–1.05)	0.69 (0.31–1.50)	0.102

Model 1: age, sex (total), current smoking, regular exercise, alcohol intake; Model 2: Model 1 + baseline body mass index, high sensitive c-reactive protein, low density lipoprotein cholesterol, HOMA-IR (log transformed), follow-up body mass index. FU, follow-up; HR, hazard ratio.

## Supplementary Materials

The following are available online at https://www.mdpi.com/2077-0383/8/5/599/s1, Table S1. Baseline characteristics of participants according to median of plasma adiponectin at baseline and change of plasma adiponectin during follow up period according to sex (A) Men, (B) Woman. Table S2. Adiponectin to leptin ratio levels according to development of metabolic syndrome.
