# Supplementary material for: Prospective Study of Sex-Specific Adiponectin Changes and Incident Metabolic Syndrome: The ARIRANG Study
Source: J Clin Med. 2019 May 1;8(5):599. doi: 10.3390/jcm8050599 (PMC6571573; doi:10.3390/jcm8050599)
Supplement: Supplementary file 1 [file jcm-08-00599-s001.pdf]

Table S1. Baseline characteristics of participants according to median of plasma adiponectin at baseline and change of plasma adiponectin during follow up period according to sex

(A) Men

Variable	Low adiponectin at baseline: Decreased adiponectin during follow-up (N=136)	Low adiponectin at baseline: Increased adiponectin during follow-up (N=93)	High adiponectin at baseline: Decreased adiponectin during follow-up (N=169)	High adiponectin at baseline: Increased adiponectin during follow-up (N=60)	p-value
**Age (years)**	55.18±8.11‡§	57.22±7.56	58.44±7.86*	58.71±7.45*	0.0015
**BMI (kg/m^2)**	23.75±2.42	23.49±2.50	23.15±2.62	23.57±3.31	0.2505
**BMI change**	-0.60±1.18†	-0.03±1.21*	-0.36±1.12	-0.22±1.24	0.0035
**Waist circumference**	84.50±6.19	83.26±6.99	83.07±6.91	83.93±7.68	0.2951
**Systolic BP (mmHg)**	125.38±16.40	126.23±16.12	126.62±15.25	126.77±14.97	0.9049
**Diastolic BP (mmHg)**	82.09±10.43	82.56±9.82	83.04±10.08	83.23±10.04	0.8322
**hsCRP (mg/L)**	0.94 (0.52-1.92)†	0.62 (0.34-1.38)*	0.81 (0.44-1.95)	0.77 (0.48-2.27)	0.0260
**BUN (mg/dL)**	16.67±4.32	15.81±4.23	16.87±4.36	16.93±4.91	0.2626
**Cr (mg/dL)**	1.08±0.14	1.06±0.13	1.06±0.15	1.09±0.17	0.3449
**Fasting glucose (mg/dL)**	95.46±17.92	94.61±12.88	92.88±16.31	93.88±22.73	0.6152
**Total cholesterol (mg/dL)**	200.48±33.38	195.70±39.18	200.70±33.94	196.75±34.10	0.6330
**HDL-C (mg/dL)**	46.99±9.63‡	47.02±10.23‡	51.18±13.96*†	51.27±10.23	0.0019
**LDL -C(mg/dL)**	116.93±30.08	117.82±32.57	115.62±29.54	117.15±30.88	0.9491
**Triglyceride (mg/dL)**	128.0 (100.0-165.0)†‡§	109.0 (83.0-136.0)*	112.0 (82.0-144.0)*	95.5 (70.0-129.0)*	0.0006
**Albumin (g/dL)**	4.75±0.28†§	4.61±0.29*	4.68±0.28	4.58±0.22*	<.0001
**Adiponectin (baseline)**	5.90 (4.74-7.20)‡§	5.65 (4.33-6.98)‡§	12.12 (10.21-14.81)*†	11.23 (10.09-13.97)*†	<.0001
**Adiponectin (follow-up)**	3.97 (2.82-5.26))†‡§	7.68 (6.16-10.73)*§	8.16 (5.77-10.48)*§	14.62 (11.68-16.61)*†‡	<.0001
**Leptin**	2.04 (1.42-3.21)	1.70 (1.20-2.56)	1.82 (1.20-2.70)	1.77 (1.28-2.51)	0.1998
**HOMA-IR**	1.48 (1.21-1.87)	1.47 (1.16-1.82)	1.36 (1.09-1.66)	1.37 (0.99-1.87)	0.1160
**Alcohol intake (%)**	103 (75.74)	60 (64.52)	111 (65.68)	41 (68.33)	0.2024
**Regular exercise (%)**	32 (24.06)	18 (19.35)	42 (25.00)	17 (28.33)	0.6128
**Current smoker (%)**	69 (50.74)	34 (36.56)	55 (32.54)	16 (26.67)	0.0019

(B) Women

Variable	Low adiponectin at baseline: Decreased adiponectin during follow-up (N=198)	Low adiponectin at baseline: Increased adiponectin during follow-up (N=128)	High adiponectin at baseline: Decreased adiponectin during follow-up (N=232)	High adiponectin at baseline: Increased adiponectin during follow-up (N=94)	p-value
**Age (years)**	51.53±7.34‡§	52.87±7.75	53.71±8.35*	55.43±8.32*	0.0007
**BMI (kg/m²)**	24.17±2.94§	24.02±2.75§	23.99±2.90§	22.78±2.83*†‡	0.0010
**BMI change**	-0.13±1.16	0.03±1.16	-0.25±1.13	-0.27±1.21	0.1338
**Waist circumference**	79.22±7.50§	77.82±7.09	79.43±8.22§	76.29±7.39*‡	0.0037
**Systolic BP (mmHg)**	118.45±15.59	121.48±16.13	121.65±16.84	123.81±17.05	0.0462
**Diastolic BP (mmHg)**	77.82±10.46	78.91±11.90	78.94±11.44	81.16±11.67	0.1350
**hsCRP (mg/L)**	0.63 (0.31-1.29)	0.63 (0.31-1.39)	0.53 (0.29-1.26)	0.49 (0.28-1.31)	0.6163
**BUN (mg/dL)**	14.40±3.58	14.02±3.65	14.91±3.79	15.36±4.53	0.0339
**Cr (mg/dL)**	0.86±0.08	0.86±0.09	0.85±0.09	0.87±0.17	0.3607
**Fasting glucose (mg/dL)**	89.25±18.19	87.45±7.82	88.11±8.07	86.97±7.23	0.3898
**Total cholesterol (mg/dL)**	200.53±37.04	196.33±33.03	204.24±37.82	199.50±39.16	0.2598
**HDL-C (mg/dL)**	48.50±10.09‡	49.60±10.88	52.41±11.29*	50.09±10.47	0.0019
**LDL-C (mg/dL)**	118.35±30.21	117.01±27.95	119.54±31.59	118.11±33.39	0.8992
**Triglyceride (mg/dL)**	98.5 (73.0-134.0)	96.0 (71.0-123.0)	94.0 (72.5-127.5)	92.0 (71.0-117.0)	0.3731
**Albumin (g/dL)**	4.60±0.23	4.56±0.23	4.58±0.25	4.52±0.26	0.0473
**Adiponectin (baseline)**	8.43 (6.82-9.73)‡§	7.05 (5.30-9.27)‡§	14.61 (12.62-18.28)*†	13.84 (12.36-16.27)*†	<.0001
**Adiponectin (follow-up)**	6.19 (4.52-7.65)†‡§	10.01 (7.28-12.51)*§	10.76 (8.20-13.23)*§	17.12 (14.33-20.31)*†‡	<.0001
**Leptin**	7.72 (5.40-7.72)‡§	7.86 (5.11-10.58)	6.34 (4.23-9.39)*	6.30 (3.91-8.82)*	0.0041
**HOMA-IR**	1.57 (1.26-2.11)§	1.48 (1.25-1.89)	1.58 (1.31-1.98)§	1.33 (1.09-1.74)*‡	0.0079
**Alcohol intake (%)**	52 (26.26)	25 (19.53)	74 (31.90)	21 (22.34)	0.0571
**Regular exercise (%)**	58 (29.44)	42 (32.81)	61 (26.52)	21 (22.34)	0.3323
**Current smoker (%)**	3 (1.52)	3 (2.34)	10 (4.31)	1 (1.06)	0.2719

BMI, body mass index; BP, blood pressure; hsCRP, high sensitive c reactive protein; HDL-C, high density

lipoprotein cholesterol; LDL-C, low density lipoprotein cholesterol; HOMA-IR, homeostasis model assessment for insulin resistance

* p < 0.05 vs. Low adiponectin at baseline & Decreased adiponectin during follow-up
† p < 0.05 vs. Low adiponectin at baseline & Increased adiponectin during follow-up
‡ p < 0.05 vs. High adiponectin at baseline & Decreased adiponectin during follow-up
§ p < 0.05 vs. High adiponectin at baseline & Increased adiponectin during follow-up

Table S2. Adiponectin to leptin ratio levels according to development of metabolic syndrome

			Non-metabolic syndrome	Incident metabolic syndrome	**p-value***
Total	Number of cases		900	167	
	Leptin	Median(Q1-Q3)	4.15 (1.92-7.73)	5.75 (2.67-9.45)	<.0001
	Leptin/Adiponectin Ratio		0.41 (0.20-0.78)	0.64 (0.32-1.16)	0.0002
Men	Number of cases		65	375	
	Leptin	Median(Q1-Q3)	1.76 (1.20-2.64)	2.45 (1.64-3.46)	0.0013
	Leptin/Adiponectin Ratio		0.20 (0.13-0.37)	0.31 (0.19-0.60)	0.0002
Women	Number of cases		525	102	
	Leptin	Median(Q1-Q3)	6.61 (4.41-9.51)	8.64 (5.88-13.87)	<.0001
	Leptin/Adiponectin Ratio		0.61 (0.35-1.05)	0.90 (0.52-1.54)	<.0001

† p-values are calculated by Wilcoxon rank-sum test
